# Comparison of Obstetric Outcomes Between Controlled-Release Dinoprostone Vaginal Delivery System (PROPESS) and Administration of Oral Dinoprostone for Labor Induction in Multiparous Women at Term

**DOI:** 10.7759/cureus.40907

**Published:** 2023-06-24

**Authors:** Nobuko Yokoyama, Shunji Suzuki

**Affiliations:** 1 Obstetrics and Gynecology, Japanese Red Cross Katsushika Maternity Hospital, Tokyo, JPN; 2 Obstetrics and Gynecology, Nippon Medical School, Tokyo, JPN

**Keywords:** multiparous women, induction of labor, oral administration, controlled-release dinoprostone vaginal delivery system (propess), dinoprostone

## Abstract

Objective: The aim of this study was to compare the rate of vaginal delivery and adverse outcomes of a controlled-release dinoprostone vaginal delivery system (PROPESS) and the administration of oral dinoprostone for labor induction in multiparous women at term.

Methods: This retrospective case-controlled study included 92 multiparous pregnant women (46 and 46 in the PROPESS and oral dinoprostone groups, respectively) who required labor induction at ≥37 weeks of gestation. The primary outcome was the success rate of vaginal delivery following the insertion of PROPESS only or the administration of oral dinoprostone (up to six tablets) only. The secondary outcomes were uterine tachysystole with non-reassuring fetal status, the proportion of cases requiring pre-delivery oxytocin, and the rate of cesarean delivery.

Results: The proportion of pregnant women who delivered vaginally as the primary outcome was significantly higher in the PROPESS group (33/46 [72%]) than in the oral dinoprostone group (16/46 [35%], p < 0.01). In the secondary outcomes, the proportion of cases requiring pre-delivery oxytocin in the PROPESS group was significantly lower than that in the oral dinoprostone group (24% vs. 57%, p < 0.01).

Conclusions: In multiparous women at term, PROPESS may be able to induce labor and lead to a higher vaginal delivery rate without adverse outcomes compared to oral dinoprostone.

## Introduction

In term-pregnant women requiring labor induction with an unfavorable cervix, cervical ripening is usually the first step. Prostaglandin E2 (PGE2) (dinoprostone) has been used as one of the methods for cervical ripening, and it has been available in different formulations of administration [1-3]. Formerly in Japan, the administration of oral dinoprostone had been widely used for labor induction and cervical ripening [4,5]; however, there has been an impression of uncertainty in its effectiveness and adjustability. In January 2020, controlled-release dinoprostone vaginal delivery systems (PROPESS, Ferring Pharmaceuticals, Saint-Prex, Switzerland) were approved by the Japan Ministry of Health, Labor, and Welfare [6].

However, wide variability in the efficiency of PROPESS has also been reported among pregnant women despite its clinical effectiveness [6-8]. In addition, there have been various recent studies to compare the efficiency of PROPESS and a metreurynter for labor induction [9]; however, to date, the comparison of obstetric outcomes between PROPESS and the administration of oral dinoprostone has not been studied.

Based on these backgrounds, the aim of the current study was to evaluate the rate of vaginal delivery and adverse outcomes of PROPESS in women requiring labor induction compared with that of administration of oral dinoprostone.

## Materials and methods

This study was approved by the Ethics Committee of the Japanese Red Cross Katsushika Maternity Hospital.

This retrospective case-controlled study analyzed data obtained from pregnant women who delivered at the Japanese Red Cross Katsushika Maternity Hospital at term. We examined multiparous pregnant women requiring labor induction at >37 weeks of gestation who had a singleton fetus in cephalic presentation and a Bishop score ≤ 6 at the start of induction between April 2020 and March 2023. In this study, we examined multiparous women only for statistical comparison although PROPESS and oral dinoprostone have also been used for nulliparous women in actual clinical practice because the cervical ripening rate has differed significantly between primiparous and multiparous women. In addition, in this study, we excluded women who are in labor, had previous uterine surgery, and had fetal heart rate (FHR) abnormalities before the intervention.

During the study period, 46 women were treated with PROPESS (Ferring Pharmaceuticals, Saint-Prex, Switzerland) after providing informed consent (PROPESS group). The PROPESS was placed in the posterior vaginal fornix with monitoring for FHR and uterine activity during insertion. If 12 hours had elapsed after insertion, the PROPESS was removed regardless of whether cervical ripening had occurred. The criteria for removal of PROPESS included (1) uterine contractions every three minutes for 30 minutes, (2) tachysystole, (3) spontaneous rupture of the membranes or performing artificial rupture of the membranes, (5) non-reassuring fetal status, and (6) appearance of mother's indefinite complaints. The following 46 women who were closest to their date of delivery and shared the same age, parity, gestational age, maternal body size, and neonatal birthweight within a 5% margin of error were selected as the oral dinoprostone group; they were administered with 0.5 mg of oral dinoprostone tablet (prostaglandin E2 [PGE2]; Prostarmon-E, Ono, Japan) every one hour until the cycle of labor pains became <5-10 minutes (up to six tablets max). After the cervical ripening treatments, oxytocin augmentation was started if contractions or progress of labor was considered inadequate in both groups.

The primary outcome was the success rate of vaginal delivery following the insertion of PROPESS only or the administration of oral dinoprostone (up to six tablets) only. The secondary outcomes were uterine tachysystole with non-reassuring fetal status, the proportion of women who required pre-delivery oxytocin, the rate of cesarean delivery, and neonatal outcomes. Neonatal outcomes included one-minute and five-minute Apgar scores as well as umbilical artery blood pH ≤ 7.1.

Data are presented as mean ± SD or number (percentage). Statistical analyses were performed using SAS version 8.02 (SAS Institute, Cary, NC, USA). A chi-square test, Fisher's exact test, or unpaired t-test was used for statistical analyses, and a p-value of <0.05 was considered significant.

## Results

Table 1 shows the clinical characteristics of the PROPESS and oral dinoprostone groups. There were no significant differences between the two groups in these variables.

**Table 1:** Clinical characteristics of the PROPESS and oral dinoprostone groups Data are presented as mean ± SD or numbers (percentages). PROPESS: Controlled-release dinoprostone vaginal delivery system.

	PROPESS group	Oral dinoprostone group	P-value
Total number	46	46	
Prity			
1	38 (83)	38 (83)	
2	8 (17)	8 (17)	1
Maternal age			
Average	34 ± 5	34 ± 5	1
≥35 years	29 (63)	29 (63)	1
Gestational age			
Average	40 ± 1	40 ± 1	1
≥41 weeks	26 (57)	26 (57)	1
Maternal height			
Average (cm)	156 ± 5	156 ± 5	1
Neonatal birth weight			
Average (g)	3114 ± 331	3099 ± 362	0.22
≥3,500 g	7 (15)	8 (17)	0.78

Table 2 shows the obstetric outcomes of the PROPESS and oral dinoprostone groups. As the primary outcome, the proportion of pregnant women who delivered vaginally following the insertion of PROPESS only was 33/46 (72%), which was significantly higher than that in the cases following the administration of oral dinoprostone only (16/46 [35%]; p < 0.01). About 33 (out of 46) women who used PROPESS had a vaginal delivery in an average of about nine hours after the insertion. In the secondary outcomes, the proportion of cases requiring pre-delivery oxytocin in the PROPESS group was significantly lower than that in the oral dinoprostone group (24% vs. 57%, p < 0.01). There were no significant differences between the two groups in other secondary outcomes as shown in Table 2.

**Table 2:** Obstetric outcomes of the PROPESS and oral dinoprostone groups Data are presented as numbers (percentages). PROPESS: Controlled-release dinoprostone vaginal delivery system; PGE2: Prostaglandin E2.

	PROPESS group	Oral dinoprostone group	P-value
Total number	46	46	
Vaginal delivery following PGE2 only	33 (72)	16 (35)	<0.01
Tachysystole and non-reassuring fetal status	1 (2)	0 (0)	0.32
Oxytocin use	11 (24)	26 (57)	<0.01
Emergent cesarean delivery	3 (7)	2 (4)	0.65
Neonatal Apgar score < 4 at 1 min	0 (0)	0 (0)	1
Neonatal Apgar score < 7 at 5 min	0 (0)	0 (0)	1
Umbilical artery pH < 7.1	0 (0)	1 (2)	0.32
Umbilical artery pH < 7.0	0 (0)	0 (0)	1

## Discussion

To the best of our knowledge, this is the first study to compare PROPESS and oral dinoprostone (PGE2) in the world, according to our PubMed search results.

Based on the current results in the multiparous pregnant women at term, the proportion of women who delivered vaginally following the insertion of PROPESS only was more significant than the proportion of women who delivered vaginally following the administration of oral dinoprostone only. The proportion of cases requiring pre-delivery oxytocin following the insertion of PROPESS only was significantly lower than that in the administration of oral dinoprostone only. Although there was no difference in final delivery or neonatal outcomes between the two methods of PGE2 administration, multiparous women using PRESS (vaginal PGE2) seemed to be more likely to reach vaginal delivery earlier than women using oral dinoprostone (oral PGE2). Therefore, the use of PROPESS may shorten the length of hospital stay in multiparous women requiring labor induction and is expected to bring benefits to the mothers and their families.

Although PGs are produced throughout the body from arachidonic acid via the cyclooxygenase pathway, PGE2 is mainly produced locally in the amniotic membrane during labor, and it locally ripens the cervix and induces uterine contractions [10]. PGs seemed to have various effects in different sites of the maternal body leading to unwanted side effects when used [10,11]. In addition, the lungs had been suggested to inactivate PGE2 in pulmonary circulation [12]. Therefore, the use of vaginal preparations for induction of labor has been reported to lessen the side effects than other routes of administration. In this study, although maternal systemic side effects could not be examined, the results suggested that intravaginal topical administration of PGE2 had a stronger effect on the induction of labor in the pregnant uterus than oral systemic administration.

We understand the presence of some serious limitations in this retrospective study. First, we cannot deny that the number of cases may be too small. We observed no significant between-group differences in the low incidence of uterine tachysystole or neonatal asphyxia, which are possible adverse events of the PGE2 preparations. Recently, the composite adverse perinatal outcome was reported to be higher in women who used vaginal PGE2 preparations than in those who used balloon catheters in labor [13]. The risk of labor with vaginal PGE2 preparations has been suggested to be associated with excessive uterine activity such as tachysystole [14]. Fortunately, tachysystole was a problem in only one case (2%) among the subjects in this study. Although vaginal PGE2 preparations may be one of the effective methods for labor induction, this risk should be kept in mind [15]. In addition, regarding PGE2 preparations used during labor, there have been some reports that it causes serious complications in mothers and fetuses although the incidence is low [10]. However, there were no cases with complications due to the small sample size in this study. In addition, PROPESS may differ from PGE2 vaginal tablets in that it can be withdrawn midway [15]. Second, we also could not examine the differences in the effect of the absorption amount of PGE2, the effect of PGE2 on systemic circulation, or the amount of PGE2 content or release between the oral and vaginal preparations. The possibility of inter-experimenter bias also cannot be denied in the pelvic examination findings that were consistent between the two groups. Therefore, a large prospective study may be needed considering the efficacy and risk of various PGE2 preparations; however, a large price difference between the two preparations (PROPESS about $150 versus oral dinoprostone about $20 per six tablets) may hinder the further consideration.

## Conclusions

In multiparous pregnant women at term, PROPESS may be able to induce labor and lead to a higher vaginal delivery rate without adverse outcomes compared to oral dinoprostone (PGE2). Therefore, the use of PROPESS may shorten the length of hospital stay in multiparous women requiring labor induction.
